# Expression of CD133 and CD44 in glioblastoma stem cells correlates with cell proliferation, phenotype stability and intra-tumor heterogeneity

**DOI:** 10.1371/journal.pone.0172791

**Published:** 2017-02-27

**Authors:** Daniel V. Brown, Gulay Filiz, Paul M. Daniel, Frédéric Hollande, Sebastian Dworkin, Stephanie Amiridis, Nicole Kountouri, Wayne Ng, Andrew P. Morokoff, Theo Mantamadiotis

**Affiliations:** 1 Department of Pathology, University of Melbourne, Melbourne, Victoria, Australia; 2 Department of Physiology, Anatomy and Microbiology, La Trobe University, Melbourne, Victoria, Australia; 3 Department of Surgery (RMH), University of Melbourne, Melbourne, Victoria, Australia; 4 Department of Medicine (RMH), University of Melbourne, Melbourne, Victoria, Australia; University of Florida, UNITED STATES

## Abstract

Glioblastoma (GBM) is a heterogeneous tumor of the brain with a poor prognosis due to recurrence and drug resistance following therapy. Genome-wide profiling has revealed the existence of distinct GBM molecular subtypes that respond differently to aggressive therapies. Despite this, molecular subtype does not predict recurrence or drug resistance and overall survival is similar across subtypes. One of the key features contributing to tumor recurrence and resistance to therapy is proposed to be an underlying subpopulation of resistant glioma stem cells (GSC). CD133 expression has been used as a marker of GSCs, however recent evidence suggests the relationship between CD133 expression, GSCs and molecular subtype is more complex than initially proposed. The expression of CD133, Olig2 and CD44 was investigated using patient derived glioma stem-like cells (PDGCs) in vitro and in vivo. Different PDGCs exhibited a characteristic equilibrium of distinct CD133+ and CD44+ subpopulations and the influence of environmental factors on the intra-tumor equilibrium of CD133+ and CD44+ cells in PDGCs was also investigated, with hypoxia inducing a CD44+ to CD133+ shift and chemo-radiotherapy inducing a CD133+ to CD44+ shift. These data suggest that surveillance and modulation of intra-tumor heterogeneity using molecular markers at initial surgery and surgery for recurrent GBM may be important for more effective management of GBM.

## Introduction

Although GBM is a relatively rare type of cancer, it has a five year survival of less than 5%, rendering it one of the most lethal types of tumors [1]. The current standard of post-surgery care is radiotherapy, in combination with the oral chemotherapeutic, temozolomide (TMZ) [2,3]. Due to the diffuse nature of GBM, complete resection of the tumor is difficult and residual malignant cells invariably cause relapse [4]. Another cause of this relapse has been suggested to be due to the presence of glioblastoma stem cells (GSCs) [5,6]. GSCs can be prospectively isolated based on the expression of the membrane associated glycoprotein CD133, which is encoded for by the *prominin-1* (*PROM1*) gene. However there are conflicting reports on the suitability of CD133 as a GSC marker [7–9], since CD133 is differentially glycosylated, leading to variable epitope masking. Another putative GSC marker is CD44, which is a ligand of hyaluronic acid (HA), a major component of the extracellular matrix [10,11].

Based on gene expression profiling of patient tumours, The Cancer Genome Atlas (TCGA) analysis classifies GBM into several molecular subtypes with the Proneural (PN) and Mesenchymal (MES) subtypes having the most distinct signatures [12,13]. Despite activation of different biological networks, there is no significant difference in long-term patient survival between these two molecular subtypes [14]. Patient derived glioma stem-like cells (PDGCs) grown *in vitro* exhibit a similar molecular classification to the parental tumor from which they originate, with two dominant cell types representing the PN and MES subtypes [15–18]. Our previous work analyzing a panel of GSC markers showed that gene coexpression modules characteristic of the GSC markers CD133 or oligodendrocyte lineage transcription factor 2 (OLIG2) were enriched in PN tumors, while a CD44 gene coexpression module was enriched in MES tumors. Cells expressing CD133 were more proliferative, cells expressing CD44 were more invasive [19] and differential expression of CD133/Olig2 or CD44 predicts response to radiotherapy [18,20,21]. More recently, genome-wide analysis of different regions within the same tumor or single cells derived from the same tumor demonstrated that multiple molecular subtypes exist in the same tumor mass [22,23] and there appears to be a stable tumor-specific equilibrium with respect to the proportion of different molecular subtypes in a GBM tumor. Cytotoxic agents have been reported to shift the cellular heterogeneity equilibrium in some cases. For example, γ-radiation and TNF-α can shift this equilibrium towards a MES phenotype [17,18,21], while an induced shift towards a PN phenotype has not been reported. If a MES to PN shift could be pharmacologically induced, this would be desirable since PN cells are more sensitive to cytotoxic therapy [17,18].

In the present study, we investigated the distribution of CD133, Olig2 and CD44 expressing patient-derived GBM cells *in vitro* and *in vivo* to determine the stability of these cell subpopulations in response to environmental perturbations/challenges. The results indicate a differential stability of the CD133/Olig2 and CD44 GBM cell subpopulations with implications for the evolution of resistant subpopulations and tumor recurrence.

## Materials and methods

### Cell culture

PDGCs were isolated from primary GBM surgeries and are designated MU##. Cells were cultured as previously described [19]. Cells were sorted on a BD FACS Aria III and analyzed on a BD LSR Fortessa. FlowJo version 8.7 was used in the data analyses. Debris was gated out using FSC vs SSC. Single cells were gated using FSC-H vs FSC-W followed by SSC-H vs SSC-W. Single-stained controls were used to construct a compensation matrix for each experiment. Isotype control samples for each individual PDGC were used for setting negative control gates, which were subsequently copied to experimental samples. The isotype controls and subsequent gating were repeated for each individual experiment.

### Animals

Animals were sourced from the Animal Resources Centre, Western Australia. Mice were housed in a pathogen-free specialized small animal facility with 12 hour dark-light cycle and had free access to mouse chow and water. To generate orthotopic GBM tumors, 6–8 week old female BALB/c-nu/nu mice (five mice per PDGC, 25 mice total) received an injection of an anti-inflammatory medication (Carprofen, 5 mg/ml, 0.5 mg/100g via intraperitoneal injection (i.p.) with a 26G needle) to reduce post-surgical pain and discomfort, then anesthetized by i,p. of a mixture of ketamine (100 mg/kg) and xylazine (10 mg/kg), using a 26G needle. Mice were then placed in a stereotaxic frame and kept on a heating blanket to maintain rectal temperature at ~37°C. A 10mm incision of the skin was made using a scalpel over a single location per mouse and portions of the bone removed with a 0.45mm diameter dental drill. 5e5 PDGC cells suspended in 5μL of HBSS were injected under stereotactic guidance to a depth of 3mm using a 2G needle driven by an electronic syringe pump (5μL over 10 min). Upon completion of the injection, the skull bone was replaced with bone wax and the incision closed by holding the edges of the incision and using safil absorbable 5/0 sutures. Mice were kept warm during recovery and observed every few minutes during the immediate post-surgery period, and then twice daily over 72 hours and then daily thereafter.

The primary experimental endpoints were greater than 10% weight loss, ataxia, and seizures lasting more than 1 min more than once per day. Mice were euthanized by CO_2_ administration and observed until all muscle activity and breathing has ceased for at least 60 seconds. The paw-pinch method was used to test for any reaction to confirm death, prior to intra-cardiac perfusion with PBS, then 4% buffered PFA. No mice died before meeting the endpoints described. Brain tissue was processed for immunohistochemistry and immunofluorescence as previously described [24].

### Immunofluorescence

For combined CD44 and Olig2 staining, both primary antibodies were mixed together and incubated at 4 degrees overnight. Fluorescently conjugated secondary antibody was incubated for four hours at room temperature in a light protected environment. Slides were mounted in aqueous media containing DAPI (ProSciTech). All images were taken using a Leica DC 3000 microscope. 16-bit grayscale images were analyzed with FIJI using a custom FIJI macros available from https://github.com/dvbrown/FijiMacros. A nuclear mask of all cells was obtained by generating an outline of DAPI labelled cells using the ‘Convert to mask’ command to ‘binarize’ the image then ‘fill holes’ to improve masking of nuclei and finally ‘outline’ to label only the circumference of the nuclei. The ‘merge channels’ function was then used to create a stack of the nuclear outline image with the Olig2 and CD44 images. This stack was then flattened with the ‘Stack to RGB’ function. Object-based co-localization was performed using the ‘Cell counter plugin in FIJI’. A cell was considered to be Olig2+CD44+ if the nucleus was positive for Olig2 and the cytoplasm positive for CD44.

### Cell cycle

Live cell cycle was performed by labelling 5e5 cells with 10μM Hoechst 33342 (Cell Signaling) for 1 hour at 37 degrees. Cells were then processed for FACS analysis and labelled with CD44, CD133 and PI. The cell cycle platform of FlowJo software was used with the ‘Watson Pragmatic’ model to quantify cell cycle.

### Environmental condition assays

A hypoxic chamber was prepared by placing a GasPakTM EZ anaerobe pouch with indicator inside a GasPak EZ Large Incubation Container (BD Biosciences). Cells were incubated in the hypoxic chamber which was placed in a standard tissue culture incubator for 24 hours prior to harvesting. 50μM TMZ (Sigma-Aldrich) was added to PDGCs daily with a 50% media change [24]. For radiotherapy experiments, PDGCs were exposed to a single 6Gy dose of radiation using a Cobalt-60 source from a Theratron Phoenix 60Co Irradiator (Best Theratronics, Canada).

### Antibodies

The antibodies used in this study were α-CD44 (Dako, M708201), α-CD44-FITC (Miltenyi-Biotec, 130-098-210), α-CD133-APC (Miltenyi-Biotech, 130-098-829), α-phospho-histone H3 (Cell signaling, 9701), IgG1-FITC (Miltenyi-Biotec, 130-098-847), IgG1-APC (Miltenyi-Biotech, 130-098- 846), α-Olig2 (Millipore, ab9610), α-Mouse Alexa Fluor 488 (ThermoFisher Scientific, A11029), α-Mouse Alexa Fluor 568 (ThermoFisher Scientific, A11004), α-Rabbit Alexa Fluor 488 (ThermoFisher Scientific, A11008) and α-Rabbit Alexa Fluor 568 (ThermoFisher Scientific, A11011).

### Ethics statement

Human cell lines derived from fresh GBM tisuue and human GBM tissue samples were sourced from surgical specimens obtained by written consent, notifying patients that the tissue may be used for research. The project was approved by the Melbourne Health Human Research and Ethics Committee and the University of Melbourne Human Research Ethics Office for Research Ethics & Integrity (Project 1339751.2).

Animal experimentation described in this study was approved by the University of Melbourne and Melbourne Health Animal Ethics Committees under the project entitled “Investigating novel mechanisms and drug targets in brain cancer”, ID: 1613869.

## Results

### Olig2+ and CD44+ tumors exhibit differential growth patterns in vivo

**Fig 1:**
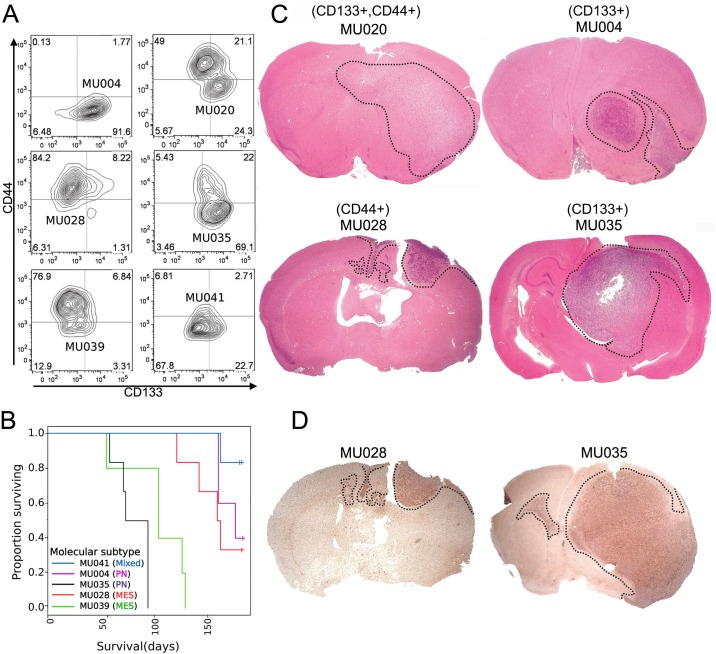
Distinct morphological growth patterns but equivalent survival of CD133+ and CD44+ classified xenografts. (A) PDGC samples were interrogated for the expression of the extracellular markers CD44 and CD133 by flow cytometry. Each individual PDGC was gated using a matched isotype control sample. (B) Lack of a survival difference between CD44+ and CD133+ classified PDGCs in an orthotopic xenograft model. 5e5 PDGC cells were injected into the cortex of BALB/c-nu/nu mice. Survival was analyzed using the Kaplan-Meier estimator method. (C) Distinct morphological growth patterns of CD133+ and CD44+ PDGCs, in vivo. Brain tissue was fixed, processed and stained with hematoxylin and eosin. Tumors are localized to the regions within the dotted lines. Arrows on MU004 and MU035 indicate the presence of white matter tract tumor cell invasion. Images were taken at 50x total magnification. (D) Similar mitotic frequency between CD44+ and CD133+ tumors in GBM xenografts. Tissue was processed for immunohistochemistry and labelled with a phospho-histone H3 antibody. Tumors are localized to the regions within the dotted lines.

Computational and functional analysis of the cell surface epitopes CD133 and CD44 has identified these markers as enriched in the PN and MES subtype of GBM respectively [17–19]. Immunophenotyping a panel of PDGCs using these markers revealed a reproducible bias in the expression of CD133 or CD44, (Fig 1A). To determine if a survival difference was associated with CD133 or CD44 bias, orthotopic xenograft models were established using five different PDGCs. A Kaplan-Meier survival analysis followed by log-rank test indicated no significant difference in survival between PDGC grafted mice. (Fig 1B). MU028 (CD44+) and MU035 (CD133+) formed the most aggressive tumors with all mice succumbing to tumours within the 6-month experimental window. Flow cytometry analysis shows that MU028 and MU035 PDGCs harbor a subpopulation of CD44+CD133+ double positive cells that are well separated from the majority of the cell population, in contrast to MU020 and MU039, where the double positive population maps closer to the majority of cells (Fig 1A).

Despite no difference in overall survival, a morphological difference in the growth patterns of CD133+ and CD44+ enriched tumors was observed (Fig 1C). Tumors generated by CD133+,CD44+ clone MU020 exhibited a large diffuse tumor, covering both left and right hemispheres, which suggests that this tumor harbors fast growing, highly invasive cells. CD133+ tumors presented as large tumors with densely packed mass of tumor cells with a circumscribed border between tumor and normal brain, exemplified by MU004 and MU035. Tumors generated by these clones also showed evidence consistent with white-matter invasion (Fig 1C arrows). CD44+ tumors, exemplified by MU028, exhibited an invasive growth pattern with multifocal infiltration of tumor cells into the normal brain parenchyma, appearing as discrete non-encapsulated clusters and swirls of densely packed tumor cell surrounded by normal brain cells. The morphological differences between xenograft tumors was reminiscent of the growth patterns of PN and MES human GBMs [17,25]. No overall difference in mitotic index between CD133+ and CD44+ xenografts was observed based on the expression of cell cycle marker, phospho-histone H3 (Fig 1D).

### GBM tumors harbor distinct OLIG2+ and CD44+ cells

**Fig 2:**
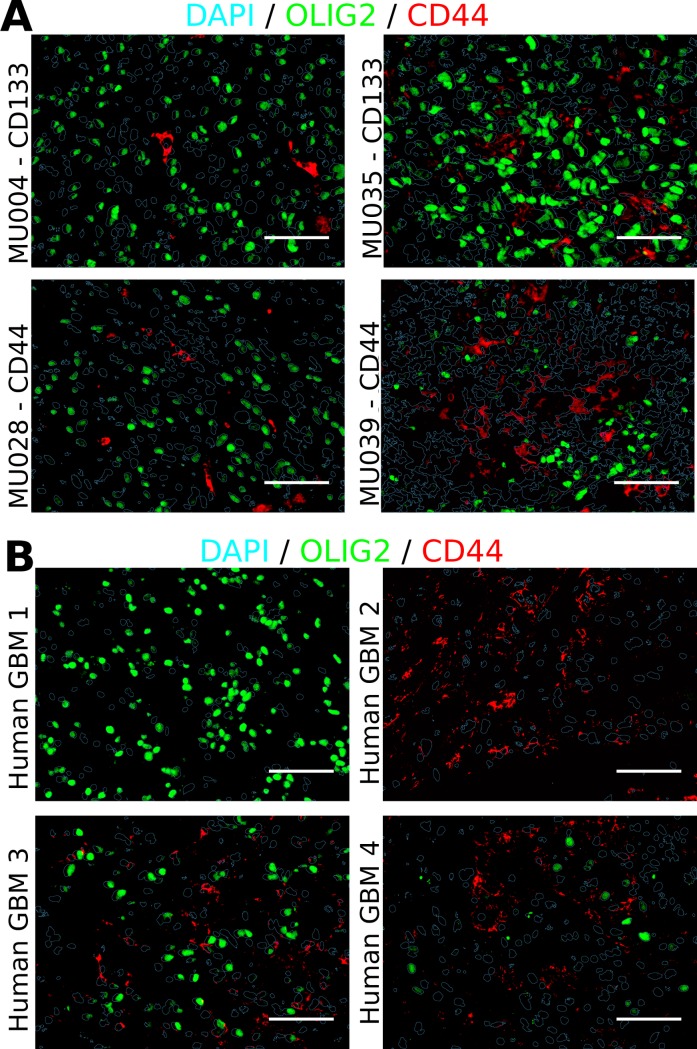
Olig2+ and CD44+ cells are distinct in human GBM. (A) Representative dual immunofluorescence of Olig2 and CD44 in PDGC xenografts. Scale bar represents 40μM. (B) Representative dual immunofluorescence of OLIG2 and CD44 in human GBM tumors. Scale bar represents 40μM.

To investigate the basis for the different tumor growth patterns observed in GBM xenografts, the distribution of OLIG2+ and CD44+ cells in the tumor tissue was investigated. For imaging analysis, DAPI was used as a nuclear mask to visualize all cells. Due to the poor immunofluorescence signal using the anti-CD133 antibody, Olig2 was used as a PN subtype marker, as previously described [17,18] (S2 Fig). The CD133+ PDGCs MU004 and MU035 generated tumors with strong Olig2 staining in the majority of cells (Fig 2A). By contrast, tumors derived from CD44+ expressing PDGCs MU028 and MU039 generated a mixture of tumor cells with discrete cells expressing Olig2+, CD44+ or neither marker.

To examine the distribution of Olig2+ and CD44+ cells in primary human GBM tumor tissue, a series of patient GBM tissue sections were examined. Similar to the human PDGC xenografts in mouse, differential GSC marker expression was observed, with some tumors predominately expressing Olig2, some predominately expressing CD44 and some with a mixture of both (Fig 2B). Using object-based co-localization, the number of cells expressing each combination of markers was quantified (Table 1). The number of cells expressing Olig2 and CD44 was found to significantly deviate from equal proportions in human GBM, consistent with a mutually exclusive expression pattern (p-value 2.2e-16, Fisher’s exact test). Interestingly, the proportion of cells expressing both markers (Olig2+CD44+) in human GBM was lower than the expected fraction (1.1% observed compared to 4.3% expected). These results indicate that the expression of CD133/Olig2 and CD44 is not random in GBM and may reflect an underlying, uncharacterized cellular state.

**Table 1:** Quantification of OLIG2 and CD44 object-based co-localization in human GBM.

	Double Negative	OLIG	CD44	Double Positive
**MU004**	714	450	36	6
**MU035**	511	271	25	9
**MU039**	768	114	275	47
**MU028**	412	168	122	22
**Human 1**	532	509	9	6
**Human 2**	475	23	178	11
**Human 3**	430	142	186	10
**Human 4**	352	112	186	8

### CD133 expressing cells are mitotically active

**Fig 3:**
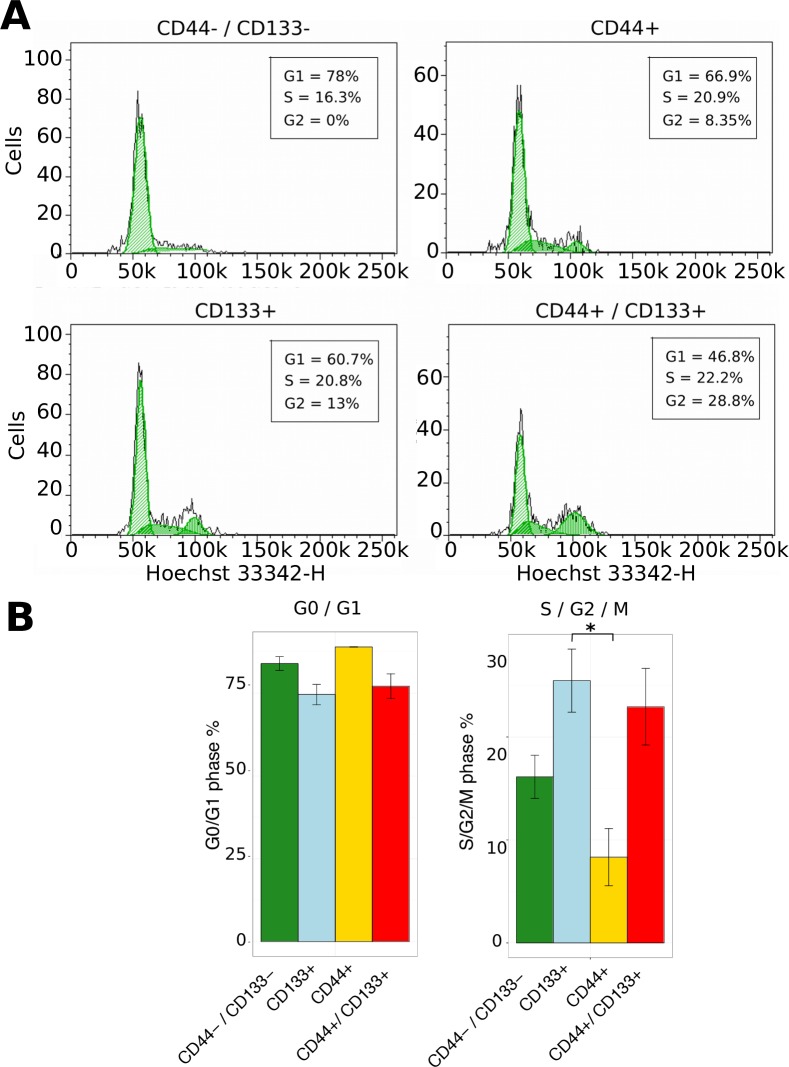
CD133+ cells are more proliferative; CD44+ cells are more quiescent. (A) Representative cell cycle analysis of CD44 and CD133 subpopulations in PDGC MU020. Live cells were pulsed with 10μM Hoechst 33342 for 60 minutes followed by immunophenotyping. Cells were gated based on CD44 and CD133 expression followed by cell cycle phase estimation. (B) Summarized cell cycle data. Error bars represent SEM of 4 distinct PDGC samples. An asterisk indicates a statistically significant difference between CD44+ and CD133+ PDGCs.

The proportion of mitotic cells in CD133+ and CD44+ GBM xenografts was not significantly different (Fig 1D). Space and nutrient limitations at the endpoint of the *in vivo* experiment may have contributed to the plateauing of proliferation and tumor expansion in all xenografts. To test this hypothesis in growth permissive conditions *in vitro*, viable PDGCs (MU004, MU020, MU035 and MU039) were pulsed with a cell permeable DNA binding dye (Hoechst 33342), followed by quantification of the surface protein expression of CD44 and CD133 by FACS. The cell cycle distribution of each subpopulation was derived by gating the four possible combinations of CD44 and CD133 marker expression and examining DNA content in each subpopulation (Fig 3A). The resolution of live S-phase cells from G1 and G2/M was not distinct enough for accurate cell cycle phase estimation compared to standard propidium iodide (PI) labelling of fixed cells (S3 Fig). Therefore we chose to group S and G2/M phases together for cell cycle quantification.

There was a significant difference in the distribution of cell cycle phases between the four subpopulations of PDGCs (p-value 0.021, one-way ANOVA under null hypothesis of equal means), (Fig 3B). The proportion of CD44-CD133+ cells in S/ G2/ M phase was significantly higher than CD44+CD133- cells (p-value 0.025, Tukey post-hoc test). A trend towards a greater proportion of CD44+CD133+ (double positive) cells in S/G2/M phase compared to CD44+CD133- cells was also observed (p-value 0.068, Tukey post-hoc test).

### The CD44+ GBM subpopulation is more stable than CD133+

**Fig 4:**
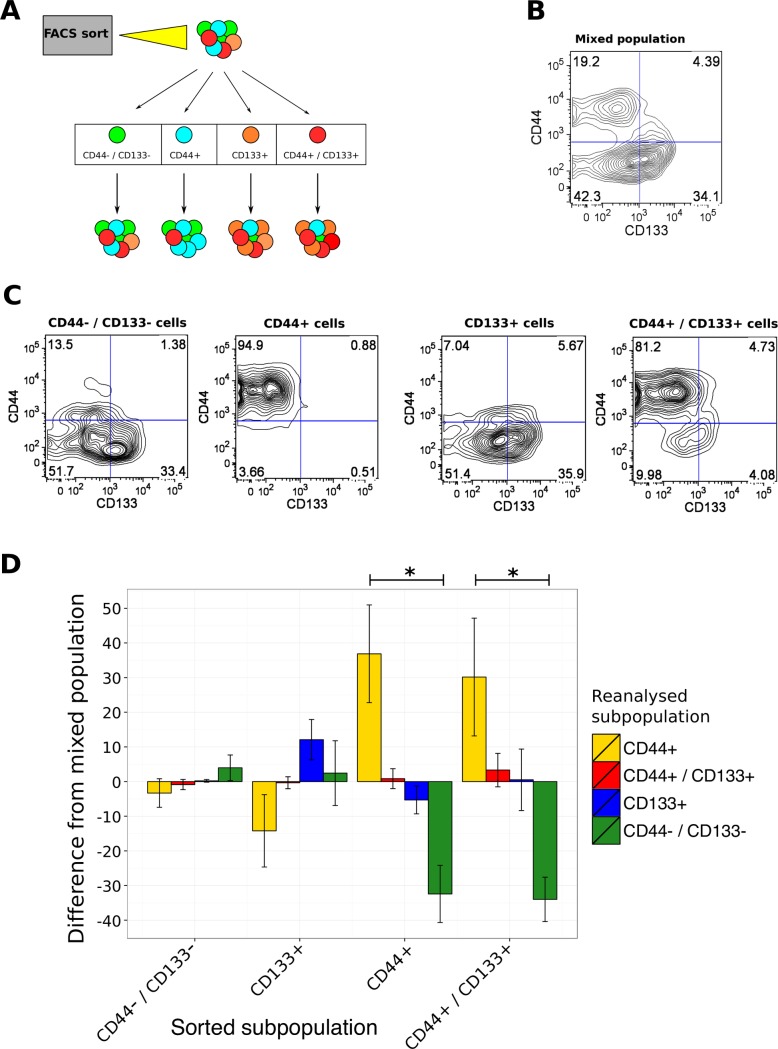
Expression of the CD44 marker *in vitro* is more stable than CD133. (A) Experimental schema of the sorting and reanalysis experiment. Gliomaspheres were sorted by FACS into pure subpopulations. Seven days later the cultures were reanalyzed for the expression of CD133 and CD44. (B) Representative FACS analysis of the MU020 unsorted control. 10,000 cells were grown for 7 days and interrogated for CD44 and CD133 expression. (C) Reanalysis of the MU020 sorted subpopulations. Sorted cells were grown for seven days and interrogated for CD44 and CD133 expression. The purified subpopulation is labelled above each plot. (D) Quantification of the sorting and reanalysis experiment. Data is presented as the % difference from the unsorted population control. Error bars represent SEM of 4 distinct PDGC samples. The initial sorted subpopulation is represented on the x-axis and the quantified subpopulation by FACS reanalysis represented by stacked bars.

To investigate the dynamics of CD133 and CD44 expression in PDGCs, a FACS sort and reanalysis experiment was performed. Mixed populations of each PDGCs (MU004, MU020, MU035 and MU039) were sorted into pure CD44-CD133-, CD44+CD133-, CD44-CD133+ and CD44+CD133+ subpopulations then reanalyzed for the expression of surface markers after seven days in culture (Fig 4A). Seven days was selected to allow for several cell divisions to occur and to enable expansion of rare subpopulations to sufficient numbers for reanalysis by FACS. To adjust for differences in the baseline distribution of CD133+ and CD44+ expressing cells in distinct PDGCs, the reanalysis data was quantified as the percent difference from the corresponding mixed population control. For example, the change in CD44 expression for representative PDGC MU020 CD44+CD133- subpopulation was 75.7% (94.9% - 19.2%) (Fig 4B and 4C).

Although there was variation in marker expression between different PDGCs, there were clear differences in the average expression across CD133 and CD44 subpopulations for the four PDGCs examined. Interestingly, the CD44+CD133- subpopulation was highly stable, retaining expression of CD44 after seven days in culture (p-value 9.72e-5, one-way ANOVA under null hypothesis of equal means) (Fig 4D). By contrast, the proportion of double negative cells was highly depleted. CD44 expression was also stable in CD44+CD133+ (double positive) cells across the four independent PDGCs (Fig 4D), (p-value 7.94e-4).

The CD44-CD133+ subpopulation did not exhibit a statistically significant deviation from equal proportions, compared to the unsorted controls (Fig 4D). There was a trend for CD44-CD133+ cells to retain expression of CD133, with a corresponding reduced proportion of CD44+ cells after seven days, although this was not significant. Cells that expressed neither marker, CD44-CD133- (double negative) resembled the unsorted control. This data indicates differential kinetics of cell surface marker phenotype, with CD44+ cells retaining CD44 expression/identity, whereas CD133+ cells exhibited variable expression. In terms of epitope stability, double positive cells behaved more like CD44+ cells and double negative cells behaved more like CD133+ cells.

### Environmental influences on CD44-CD133 equilibrium

**Fig 5:**
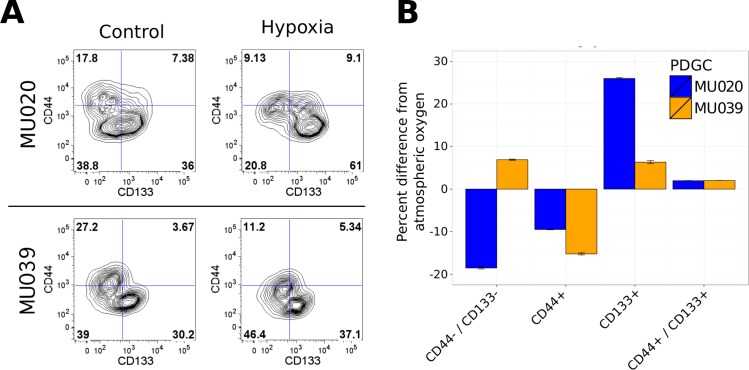
A decrease in CD44+ cells in response to hypoxia. (A) MU020 and MU039 cells were incubated for 24 hours in a hypoxic chamber, followed by immunophenotyping for CD44 and CD133 expression. (B) Changes in surface protein expression for cells grown in hypoxia were quantified as the difference from the proportion of cells expressing the marker in control (atmospheric oxygen) cells. (C) Representative FACS analysis of MU020. PDGCs were treated with a single 6Gy dose of radiation followed daily treatment with 50μM TMZ or combination therapy. Five days later cells were immunophenotyped for CD44 and CD133 expression. (D) Effect of GBM therapy on expression of CD44 and CD133. Changes in surface expression was quantified as the difference from control treated (DMSO) cells. Error bars represent SEM of 4 distinct PDGCs.

Given the relative stability of the CD44+ phenotype under basal conditions, the stability of the CD44 and CD133 PDGC subpopulations under perturbed conditions was investigated. The PDGC samples MU020 and MU039 were selected, as they harbor distinct CD44+ and CD133+ subpopulations that would facilitate the examination of changes in subpopulation frequency (Fig 5A). The influence of oxygen tension on CD44-CD133 equilibrium was explored by growing PDGCs in a hypoxic environment for 24 hours prior to quantification of surface protein expression by FACS analysis. The proportion of CD44+CD133- cells was significantly decreased (p-value 4.1e-4, pairwise t-test with FDR correction) and the proportion of CD44-/ CD133+ cells was significantly increased (p-value 7.5e-4) in MU020 (Fig 5A and 5B). The decrease in the proportion of CD44+ cells in MU039 was more pronounced in comparison under hypoxic conditions (p-value 2.46e-6) but the increase in the CD133+ subpopulation was smaller (p- value 2.2e-3). Interestingly, the proportion of double positive cells in the total population did not change significantly in both PDGCs.

**Fig 6:**
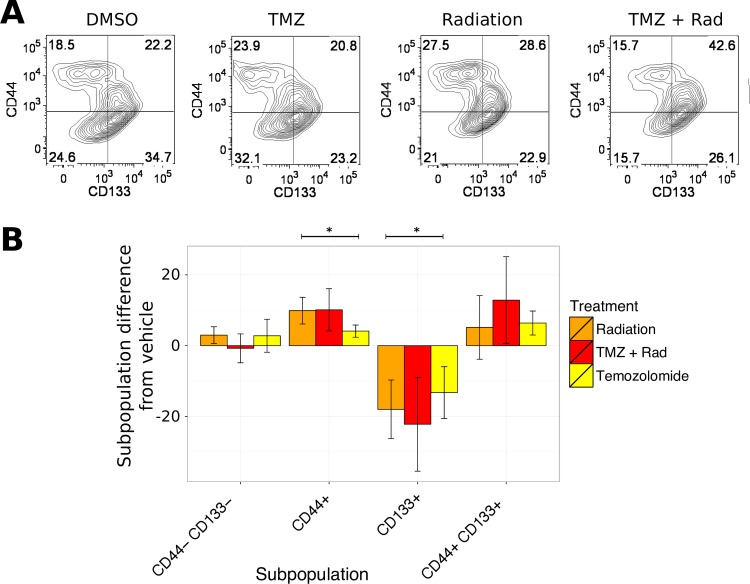
An increase in CD44+ cells in response to therapy. (A) Representative FACS analysis of MU020. PDGCs were treated with a single 6Gy dose of radiation followed daily treatment with 50μM TMZ or combination therapy. Five days later cells were immunophenotyped for CD44 and CD133 expression. (B) Effect of GBM therapy on expression of CD44 and CD133. Changes in surface expression was quantified as the difference from control treated (DMSO) cells. Error bars represent SEM of 4 distinct PDGCs (MU004, MU020, MU035 and MU039).

To explore the effect of GBM chemotherapy on CD44-CD133 equilibrium, MU004, MU020, MU035 and MU039 PDGCs were treated with sub-lethal doses of temozolomide (TMZ) and radiotherapy (Fig 6A). Sub-lethal doses were used to specifically investigate changes in surface marker expression to minimize the selection of subpopulations due to differential sensitivity to cytotoxic agents (S4 Fig).

The proportion of cells expressing CD44 significantly increased after five days of combination treatment (p-value 5.7e-3, t-test under null hypothesis of mean difference of 0) (Fig 6A and 6B). Conversely, the proportion of CD133 expressing cells significantly decreased after combination treatment (p-value, 6.5e-3). The proportion of double negative cells did not change. There was a trend for combination treatment (TMZ and radiation) to increase the proportion of double positive cells but this only apparent in PDGCs with a prominent CD133+ subpopulation before treatment (MU004 and MU035). The data indicates that environmental conditions influence/shift the proportion of cells expressing CD44 and CD133 extracellular markers.

## Discussion

This study confirms prior observations regarding the enriched expression in GBM of the extracellular markers CD44 and CD133 and the nuclear marker Olig2 [17,18]. The majority of cells *in vitro* or *in vivo* express either CD44 or CD133. The proportion of CD44+ and CD133+ subpopulations in each PDGC was unique. As the cell culture conditions and therefore microenvironment was equivalent for the four PDGCs, the cell culture specific CD133/Olig2/CD44 cellular equilibrium was likely due to the unique genetic background of the original GBM tumors. Characterization of the genomes of these patients by next generation sequencing will be necessary to clarify this. Although there was no overall survival difference observed between CD44+ and CD133+ enriched PDGCs, there was evidence of differing growth patterns, with CD133+ tumors exhibiting circumscribed growth and CD44+ tumors exhibiting invasive growth, *in vivo*. The xenograft experiment was conducted in the absence of therapeutic challenge, unlike the situation in the clinic, where patient tumors are exposed to cytotoxic therapies. Without selective pressure from therapy, the greater proliferative potential of PN cells may mask a survival difference expected between the PN and MES subtypes. The mixed PDGC, MU041 had the lowest tumorgenicity of the patient derived samples tested. This was the only sample where the majority of the cells had a double negative immunophenotype (not expressing CD133 or CD44). Although these cells were able to grow *in vitro*, this observation raises the possibility that CD44 or CD133 may be required for growth *in vivo*. Investigation of further independent PDGCs displaying a double negative immunophenotype will be required to more confidently address the link between heterogeneity and tumorigenicity in an orthotopic xenograft mouse model. Xenograft tumors derived from CD133+ PDGCs were composed of Olig2+ cells in contrast to tumors from CD44+ PDGCs, which were more heterogeneous.

The proportion of Olig2+CD44+ (double-positive) cells was lower than expected, *in vivo*. These cells were actively cycling and could give rise to the other GBM subpopulations (Figs 3 and 4). Rarity, proliferation and multipotency are all properties ascribed to cancer stem cells. We previously showed that the CD44+CD133+ PDGC subpopulation has better sphere forming potential *in vitro*, using a limiting dilution assay [19]. Further functional investigation of double-positive cells is warranted and may represent an important subpopulation to target.

CD133+ PDGCs, regardless of CD44 status, were enriched for cells in S and G2/M phase, consistent with computational and functional evidence that CD133 expression correlates with actively cycling cells [9,19,26]. This study observed no gross difference in the number or proportion of mitotic cells, *in vivo*. As our analyses on xenograft tumors were performed after mice were symptomatic, growth conditions may have been constrained by hypoxia and necrosis. Cells expressing CD133 were more likely to convert to CD44+ cells after 7 days in culture than the reverse scenario. This property may be linked to the proliferative phenotype of CD133+ cells. Analysis of hES cells with the FUCCI reporter system has revealed that cell fate choice is made in early G1 phase [27]. The ability of CD133+ cells to change their phenotype may therefore be due to their actively cycling status. PDGC cells expanded *in vitro* exhibited an average doubling time of around two to three days, similar to what we previously reported (19). A seven day timepoint for phenotype analysis was chosen to enable the expansion of rare subpopulations to sufficient numbers for reanalysis by FACS and also allowing enough time for the population to establish an equilibrium of cell states due to cell phenotype shifting. Culturing cells for longer periods led to cell clumping which may affect cell phenotype. To determine the kinetics of CD44+ to CD133+ transition, additional timepoints need to be investigated.

The correlation of CD133+ cells with proliferation observed in the present study and the correlation of CD44+ cells with invasion reported in our prior study [19] are reminiscent of the PN-MES molecular subtypes in GBM. However, the PN and MES subtypes have been defined based on global gene expression signatures, so the use of a single marker such as CD44 or CD133, although convenient, needs further validation. A limitation of our experimental approach is that we could not track the fate of each individual cell over time to definitively rule out selection of existing minor subclones. An experiment to clearly delineate between differential proliferation or viability of PN and MES cells and true plasticity would be to use a lineage tracing approach [28]. The greater variability in the proportion of CD133 expressing cells in a PDGC population over time demonstrated in this study may explain the lack of robustness of CD133 as a cancer stem cell marker.

Surprisingly, hypoxia decreased the proportion of CD44 expressing cells and increased the proportion of CD133 expressing cells. Cells found at the hypoxic tumor interface and associated the morphological feature known as pseudopalisading, decrease proliferation and activate a migratory program to escape the hypoxic core [29], indicating pseudopalisading cells may be MES-like. This is in contrast to our data with the hypoxia experiments demonstrating a decrease in CD44 expression. The hypoxic chamber used in our experiments may in fact more closely resemble the normal microenvironment of the brain, where the level of oxygen is between 0.5% and 8% [30]. Hypoxia has previously been shown to promote the proliferation of GSCs with increased expression of CD133 and acquisition of stem-like phenotypes in culture [31] consistent with acquisition of PN properties. Our interpretation of this data is that our *in vitro* experimental setup is not reflective of hypoxia experienced *in vivo* by GBM tumors. For these reasons, care should be taken in differentiating between *in vitro* hypoxia, in a cell culture system, compared to hypoxia *in vivo*. The higher free radical concentration at atmospheric oxygen levels may inhibit the growth of PDGCs in standard (5% CO_2_, ~20% O_2_) cell culture conditions.

Our work also shows that temozolomide decreases the proportion of cells expressing CD133 and increases the proportion of cells expressing CD44 and that this effect is additive with a further cytotoxic challenge, including radiation. This decrease in the proportion of proliferative cells and corresponding increase in invasive cells shares many features with Epithelial to Mesenchymal transition (EMT) in other cell types [32,33]. Our experimental design did not allow us to differentiate between EMT and CSC derived heterogeneity. However, these two models may not necessarily be distinct, with studies demonstrating that the EMT process can generate cells with CSC properties [34,35]. CD133+ and CD44+ cells with their respective proliferative and invasive properties resemble the phenomenon of phenotype switching in melanoma tumors [36,37]. These cell states are dynamic in nature as opposed to their initial description as static molecular subtypes.

## Conclusion

The data presented here highlights the importance of non-genetic acquisition of therapeutic resistance which occurs in combination with ongoing genetic selection of subclones [38,39]. Overcoming both genetic and non-genetic resistance mechanisms presents a significant challenge for future GBM therapies. The expression of CD133, Olig2 and CD44 are correlated with the proliferative or invasive state of a GBM cell. Proliferation in turn correlates with sensitivity to therapy. By monitoring the changes in cellular phenotype using CD133/Olig2 and CD44 markers in the clinic, an improved therapeutic regimen could be designed to minimize acquisition of non-genetic tumor resistance.

## Supporting information

S1 Fig Isotype control for flow cytometry (related to Fig 1). Representative isotype control for PDGC MU035. Cells were labelled with IgG1-APC and IgG1-FITC antibodies.
(TIF)

S2 Fig Immunofluorescence image processing (related to Fig 2). Individual images of GBM tumors prior to merging. Scale bar represents 100μM. (B) Isotype control of PDGC xenograft MU039. Sections were labelled with IgG1-AlexFluor488 and IgG1-AlexFluor568. (C) Screenshot of manual counting implemented in FIJI. "1" represents OLIG2-CD44- cells, "2" represents OLIG2-CD44- cells, "3" represents OLIG2-CD44+ cells and "4" represents OLIG2+CD44+ cells.
(TIF)

S3 Fig Cell cycle analysis with propidium iodide (related to Fig 3). Cell cycle analysis of MU020 was performed by fixing cells with dropwise addition of 70% ethanol. Cells were incubated overnight at -20 degrees. Cells were washed twice in PBS followed by the addition of 50μg/mL PI and 100μg/mL RNase A. The staining solution was incubated at room temperature for 30 minutes prior to FACS analysis.
(TIF)

S4 Fig Cell viability analysis of PDGCs treated with temozolomide (related to Fig 6). 50μM of temozolomide was added 72 hours prior to quantification of cell number with resazurin. Mean temozolomide sensitivity is presented relative to DMSO which was the vehicle. Error bars represent SEM of four distinct PDGCs (p = 0.947, pairwise t-test).
(TIF)

S1 Dataset Zipped primary data used to generate the figures in this study. (ZIP)
