# Supplementary figures and images for: Expression of CD133 and CD44 in glioblastoma stem cells correlates with cell proliferation, phenotype stability and intra-tumor heterogeneity
Source: PLoS One. 2017 Feb 27;12(2):e0172791. doi: 10.1371/journal.pone.0172791 (PMC5328356; doi:10.1371/journal.pone.0172791)

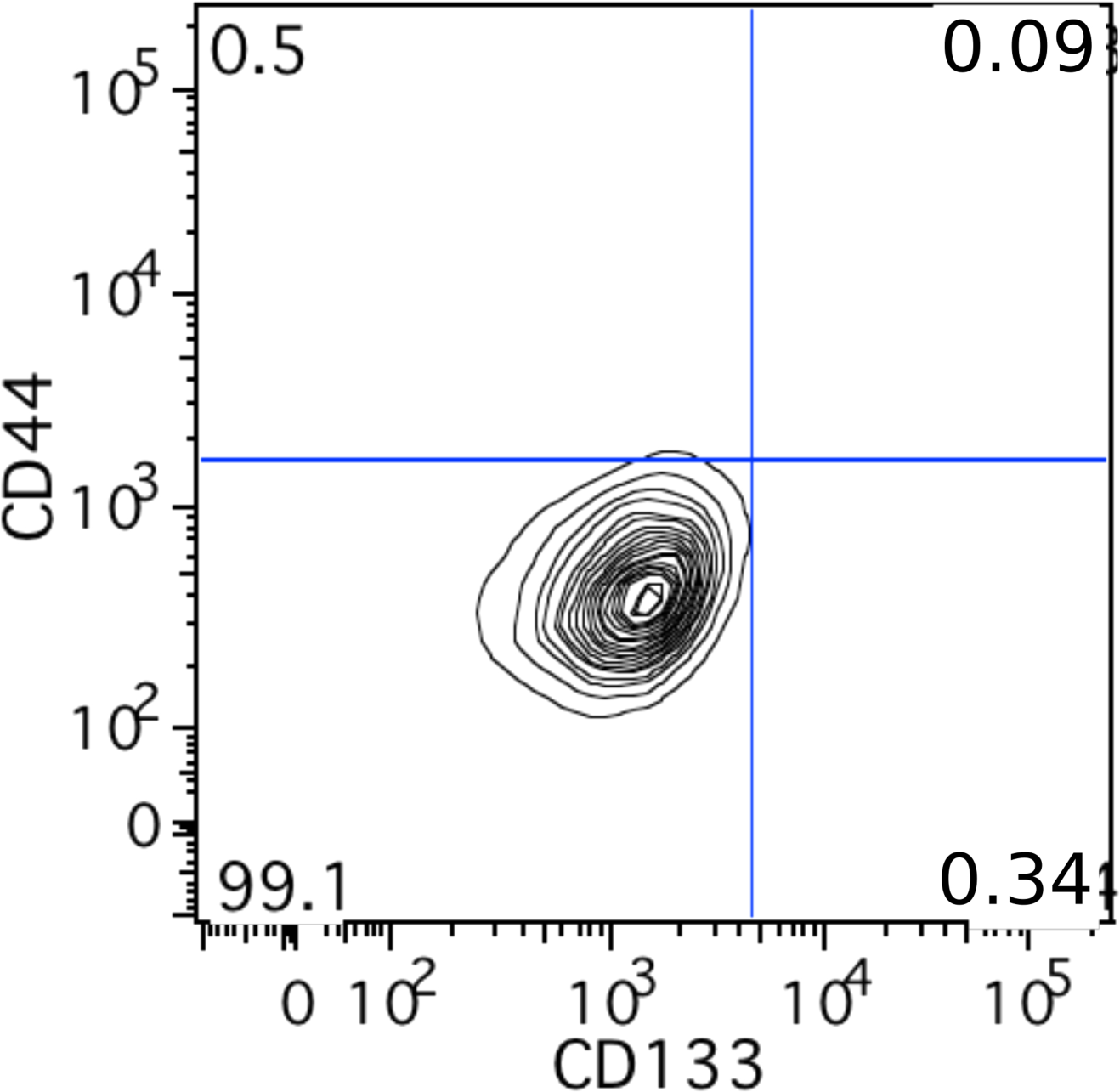

Supplement: S1 Fig — Representative isotype control for PDGC MU035. Cells were labelled with IgG1-APC and IgG1-FITC antibodies. (TIF) [file pone.0172791.s001.tif]

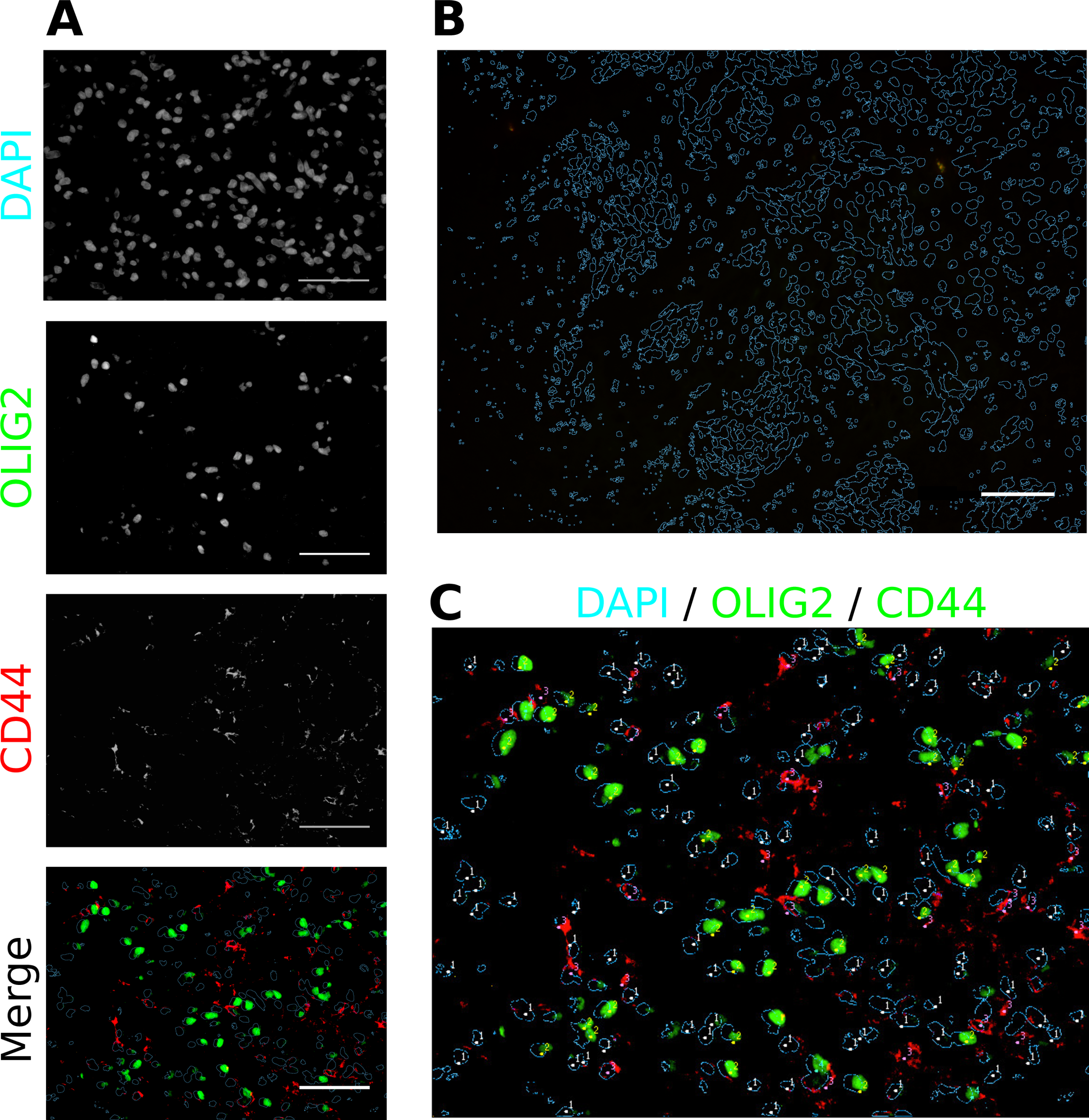

Supplement: S2 Fig — Individual images of GBM tumors prior to merging. Scale bar represents 100μM. (B) Isotype control of PDGC xenograft MU039. Sections were labelled with IgG1-AlexFluor488 and IgG1-AlexFluor568. (C) Screenshot of manual counting implemented in FIJI. "1" represents OLIG2-CD44- cells, "2" represents OLIG2-CD44- cells, "3" represents OLIG2-CD44+ cells and "4" represents OLIG2+CD44+ cells. (TIF) [file pone.0172791.s002.tif]

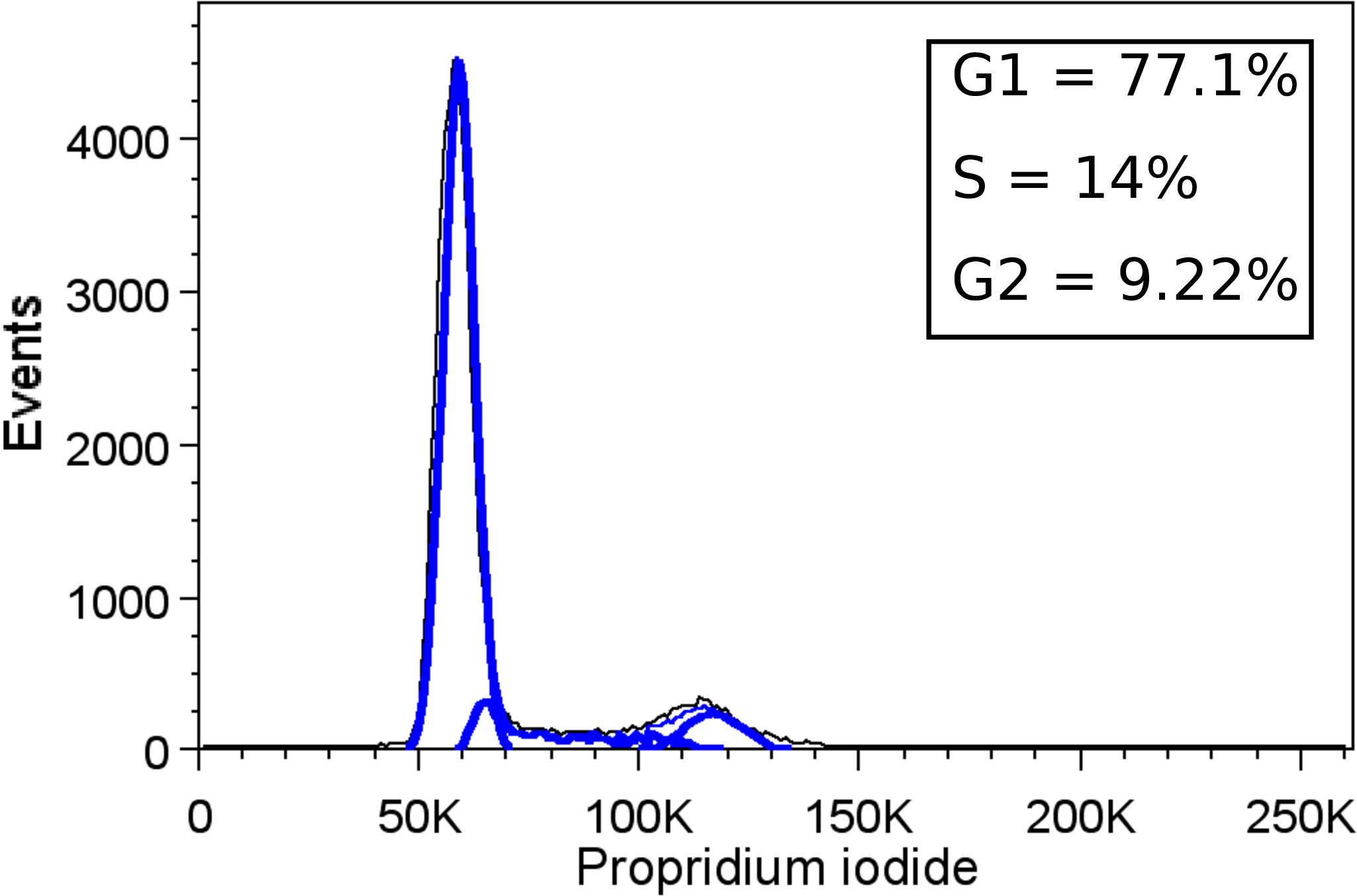

Supplement: S3 Fig — Cell cycle analysis of MU020 was performed by fixing cells with dropwise addition of 70% ethanol. Cells were incubated overnight at -20 degrees. Cells were washed twice in PBS followed by the addition of 50μg/mL PI and 100μg/mL RNase A. The staining solution was incubated at room temperature for 30 minutes prior to FACS analysis. (TIF) [file pone.0172791.s003.tif]

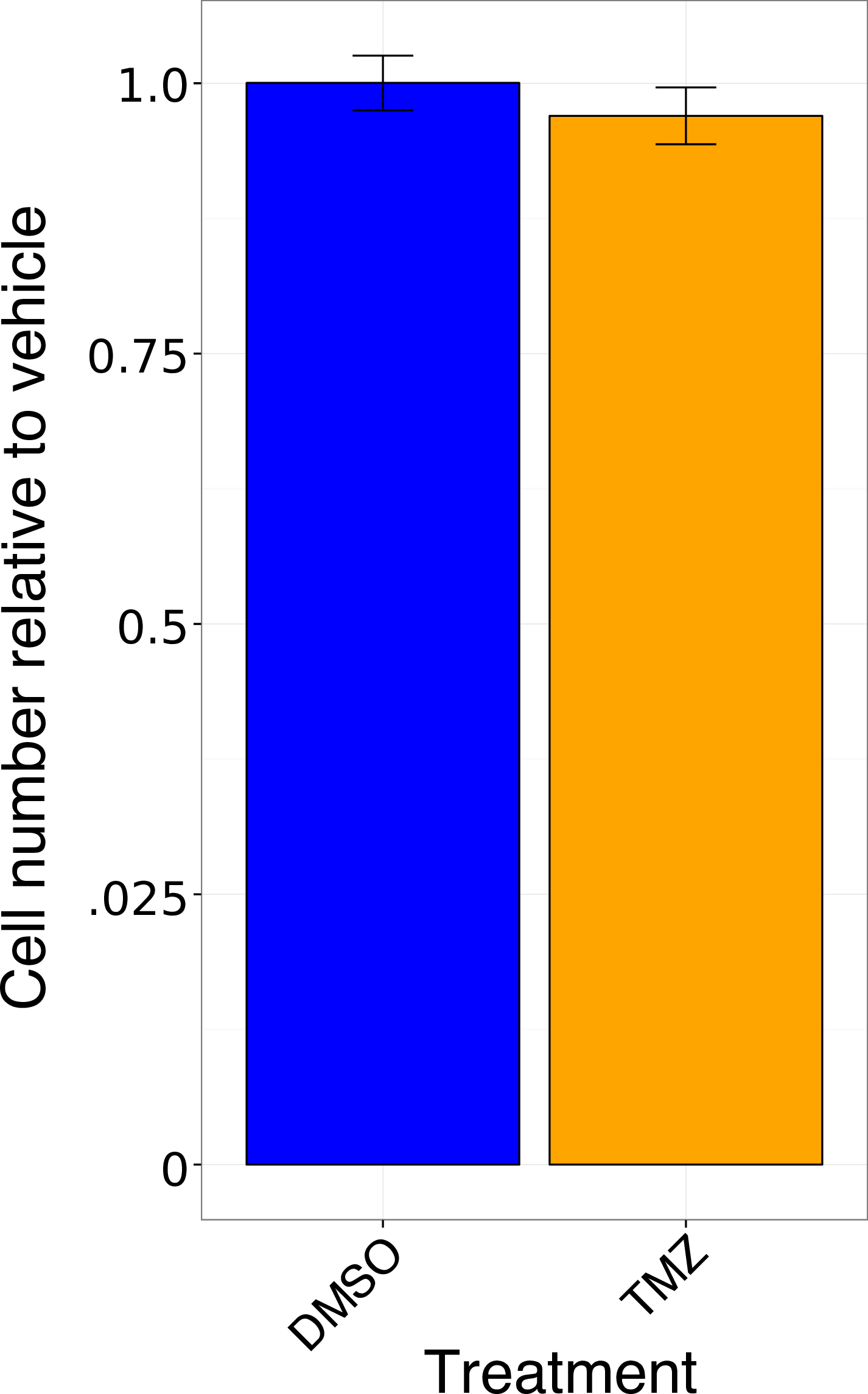

Supplement: S4 Fig — 50μM of temozolomide was added 72 hours prior to quantification of cell number with resazurin. Mean temozolomide sensitivity is presented relative to DMSO which was the vehicle. Error bars represent SEM of four distinct PDGCs (p = 0.947, pairwise t-test). (TIF) [file pone.0172791.s004.tif]
